# Pupil maximum constriction velocity predicts post-induction hypotension in patients with lower ASA status: a prospective observational study

**DOI:** 10.1186/s12871-022-01808-0

**Published:** 2022-08-31

**Authors:** Luyi Shao, Yaqing Zhou, Zichuan Yue, Zhongya Gu, Jie Zhang, Kangli Hui, Jingwei Xiong, Miaomiao Xu, Manlin Duan

**Affiliations:** 1 College of Anesthesiology, Xuzhou Medical University, Xuzhou, 221004 Jiangsu China; 2 Department of Anesthesiology, Affiliated Jinling Hospital, Medical School of Nanjing University, Zhongshan North Road #305, Nanjing, Jiangsu Province, 210002 China; 3 Department of Anesthesiology, BenQ Medical Center, The Affiliated BenQ Hospital of Nanjing Medical University, Nanjing, 210019 Jiangsu China

**Keywords:** Anesthesia induction, Post-induction hypotension, Pupillary light reflex, Maximum constriction velocity

## Abstract

**Background:**

Individuals affected by autonomic dysfunction are at a higher risk of developing hypotension following anesthesia induction. Dynamic pupillometry has previously been employed as a means of assessing autonomic function. This prospective observational study was developed to determine whether pupillary light reflex (PLR) parameters can reliably predict post-induction hypotension (PIH).

**Methods:**

This study enrolled patients with lower ASA status (I-II) undergoing elective surgery. PLR recordings for these patients prior to anesthesia induction were made with an infrared pupil camcorder, with a computer being used to assess Average Constriction Velocity (ACV), Maximum Constriction Velocity (MCV), and Constriction Ratio (CR). PIH was defined by a > 30% reduction in mean arterial pressure (MAP) or any MAP recording < 65 mmHg for at least 1 min from the time of induction until 10 minutes following intubation. Patients were stratified into PIH and non-PIH groups based on whether or not they developed hypotension.

**Results:**

This study enrolled 61 total patients, of whom 31 (50.8%) exhibited one or more hypotensive episodes. Patients in the PIH group exhibited significantly smaller ACV (*P* = 0.003) and MCV values (*P* < 0.001), as well as a higher CR (*P* = 0.003). Following adjustment for certain factors (Model 2), MCV was identified as a protective factor for PIH (Odds Ratio: 0.369). Receiver operating characteristic (ROC) analyses revealed that relative to CR (AUC: 0.695, 95% CI: 0.563–0.806; *P* = 0.004), the reciprocal of MCV (1/MCV) offered greater value as a predictor of PIH (AUC: 0.803,95%CI: 0.681–0.894; *P* < 0.001).

**Conclusion:**

These results indicate that pupil maximum constriction velocity is a reliable predictor of post-induction hypotension in individuals of ASA I-II status undergoing elective surgery.

**Trial registration:**

This study was registered with the Chinese Clinical Trial Registry (registration number: ChiCTR2200057164, registration date: 01/03/2022).

## Introduction

Intraoperative hypotension is a frequently observed complication of general anesthesia that is associated with an elevated risk of postoperative complications including acute kidney injury, heart failure, and stroke [1–3]. Such hypotension can develop at different times in the context of anesthetization through a range of mechanisms [4], with post-induction hypotension (PIH) being a particularly common form of this condition that occurs during the interval between anesthesia induction and the initiation of surgical stimulation [5]. PIH incidence is thought to be associated with the anesthesia-mediated inhibition of circulation and the lack of stimulation during the period of anesthesia induction.

Patients exhibiting autonomic dysfunction and poor blood volume are more likely to develop PIH [6, 7]. Blood volume status can be assessed through ultrasonographic measurements of the diameter of the inferior vena cava or subclavian vein and associated collapsibility indices, providing an accurate and rapid means of gauging PIH risk [7–9]. In contrast, the relationship between autonomic dysfunction and PIH remains incompletely understood, with heart rate variability (HRV) being the only generally accepted approach to assessing the autonomic nerve function of a given patient despite its high technical requirements and relatively poor ability to accurately predict PIH incidence [6, 10].

Dynamic pupillometry is a newly developed autonomic testing tool currently being evaluated to determine the association between the pupil and anesthesiological endpoints. These include evaluating nociception during general anesthesia, quantifying opioid effects, and monitoring brainstem function after cardiac arrest and traumatic brain injury [11, 12]. Pupillary light reflex (PLR) parameters have been associated with patient HRV, a method of predicting PIH, in the earlier research by Okutucu et al. [13]. At the same time, in 2019, Ryohei Miyazaki reported that maximum pupil diameter was positively correlated with PIH incidences in patients with an ASA status of I-II [14]. In light of the results mentioned above, the hypothesis that pre-operative variations in pupillary light responses can act as a reasonable predictor of PIH was generated for the present study.

To develop a new method of determining PIH risk, we measured the pre-operative pupillary parameters before induction in this prospective observational study. Then, we examined whether particular pupil parameters can act as independent predictors of PIH in individuals with lower ASA status (I-II).”

## Methods

### Patients

This prospective observational study was conducted at Jinling Hospital, which is a large Chinese tertiary-level teaching hospital. The hospital ethics committee approved this trial (2022NZKY-013-01), which was registered with the Chinese Clinical Trial Registry (registration number: ChiCTR2200057164, registration date:01/03/2022). All participating patients provided written informed consent.

Eligible patients were individuals 18–64 years of age with an ASA physical status of I-II who were scheduled to undergo elective surgery under general anesthesia. Patients were excluded if they had a history of eye diseases, eye surgery, neuromuscular dysfunction, diabetes mellitus, hypertension, thyroid dysfunction, pupil malformations, used drugs that impact pupil size, used alpha or beta-blockers, had a scheduled operative duration of < 30 minutes, were scheduled to undergo neurosurgery, or were undergoing surgical procedures in the lateral or prone position. If patients experienced failed arterial catheterization, secondary tracheal intubation, a loss of mean arterial pressure (MAP) or heart rate (HR) data, or a loss of pupil data, they were withdrawn from testing.

### Pupillary measurements

**Fig. 1:**
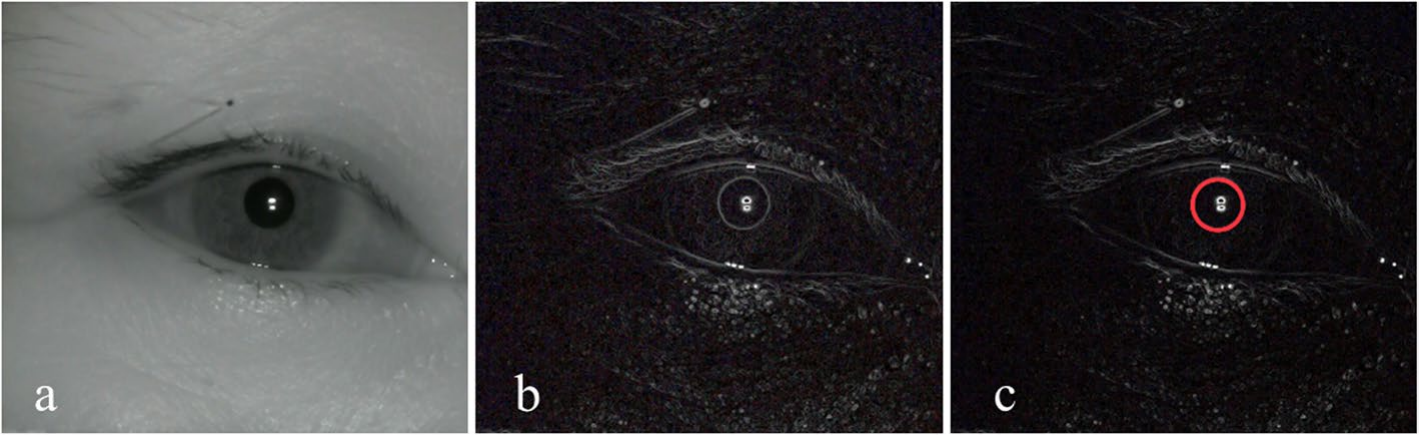
**a** PLR images decomposed by Adobe Premiere Pro; **b** The automated enhancement of image borders by ImageJ; **c** Identified pupil contours

All patients waited in the anesthetic preparation room before entering the operating room and receiving anesthesia induction. Patients were instructed to rest in the supine position for 10 minutes in the preparation room before receiving pupillary examinations. Between 9 and 12 AM, a single researcher tested all pupillary light reflex responses in order to reduce the potential effect of diurnal variation on the results. Pupillary measurements were made with an infrared pupil camcorder (LRCP 10910; resolution: 1080p; focal length: 8 mm; angle: 45° undistorted) [15, 16] that was positioned 25 cm above the right pupil and captured images at 30 frames/s. Light reflection was achieved using a yellow LED light source (color temperature: 5000 K) at an equivalent height. Camcorder video data were continuously relayed to a computer during the measurement process, with image data being processed in an automated manner using Adobe Premiere Pro, v 14.0.0 (Adobe, USA). Pupil parameters in the resultant images were measured by two researchers using ImageJ v 1.51 J8 (National Institutes of Health, USA), which was able to enhance pupillary contours in processed images in an automated manner with a 0.1 mm measurement sensitivity (Fig. 1).

After computer recordings were complete, the Baseline Pupil Diameter (BPD), Minimum Pupil Diameter (MPD), Pupil Constriction Latency (PCL), Pupil Constriction Time (PCT); Average Constriction Velocity (ACV) [$ACV=\frac{BPD- MPD}{PCT}$], Maximum Constriction Velocity (MCV), and Constriction Ratio (%) [$CR=\left(\frac{MPD}{BPD}\right)\ast 100\%$] were recorded.

### Anesthesia management

All patients fasted beginning at 10 PM on the day before the scheduled surgery, and no patients had been premedicated. All patients were subjected to electrocardiogram, respiratory rate, blood pressure, pulse, airway pressure, and end-tidal CO_2_ monitoring. Patients were initially infused with Ringer’s acetate solution (10 ml/kg/h), and induction was administered at 5 min post-arterial puncture with all patients undergoing a standard rapid sequence induction: 0.04 mg/kg midazolam, 0.3 μg/kg sufentanil, 2 mg/kg propofol and cisatracurium 0.15 mg/kg. At 3 minutes following the administration of the muscle relaxant, an experienced anesthesiologist performed endotracheal intubation using a Disposcope endoscope.

### Blood pressure measurements

All patients underwent invasive blood pressure monitoring via non-operative radial artery catheterization. Following admission to the operating room, an experienced anesthesiologist inserted a 22-gauge arterial catheter (B. Braun, Germany) into each patient, with puncture sites being selected using the 1–2 cm of the styloid process where the radial pulse was most pronounced. The catheter was then connected to a pressure sensor (B. Braun, Germany), flushed using heparinized saline, and MAP was then recorded every 1 min by the monitor (Mindray, China).

### Data collection

Patient characteristics including age, sex, weight, height, current medications, and comorbid diseases were collected from hospital records. MAP and HR were recorded every 1 min from before anesthesia induction until 10 min following tracheal intubation, with baseline MAP being defined as that taken the data recorded 1 min before induction.

PIH was defined as a > 30% reduction in MAP or any recorded MAP < 65 mmHg for at least 1 min during the interval from induction until 10 min post-intubation. Patients were separated into PIH and non-PIH groups based on whether or not they experienced any hypotensive episodes during this interval.

### Sample size calculations

Based on the results of a prior study, it was determined that 22 patients would be required in the two groups to detect differences in pupil constriction velocity, while 30 patients would be required to resolve differences in baseline pupil diameter [14]. As the incidence of PIH among 130 patients undergoing general anesthesia in our hospital was 50%, the minimum number of cases required for the construction of a logistic regression model (Model 1) was 60. To account for a dropout rate of 10%, a sample size of 67 was thus required to provide sufficient statistical power to the present study.

### Statistical analysis

Microsoft Excel (v 2202, Microsoft, USA) was used to collect data. The Kolmogorov-Smirnov test was used to assess the normality of collected data, with normally distributed results being reported as means ± standard deviation ($\overline{\mathrm x}$  ± s) and compared via independent sample t-tests, while non-normally distributed data were reported as the median [interquartile range] and compared via Mann-Whitney U tests. Categorical data were compared using chi-square tests and reported as numbers (%). Pearson correlation coefficients (r) were used to assess the association between pupil measurements and the percentage MAP decrease.

The relationship between pupillary parameters and PIH incidence was assessed using two multivariate logistic regression models. Considering a strong association between pupil parameters and age, age was included individually as a variable in model 1 [17]. While Model 2 was adjusted for other variables based on the results of our studies and other prior large-scale retrospective analyses, including age, sex, albumin levels, BMI, ASA grade, and baseline MAP [5, 18, 19]. Receiver operating characteristic (ROC) curves were then used to assess the ability of pupillary parameters to predict PIH based on the results of these analyses.

SPSS v24.0 (IBM, USA) and MedCalc v19.0 (MedCalc Software, Belgium) were used for all statistical analyses, with *P* < 0.05 as the threshold of statistical significance.

## Results

**Fig. 2:**
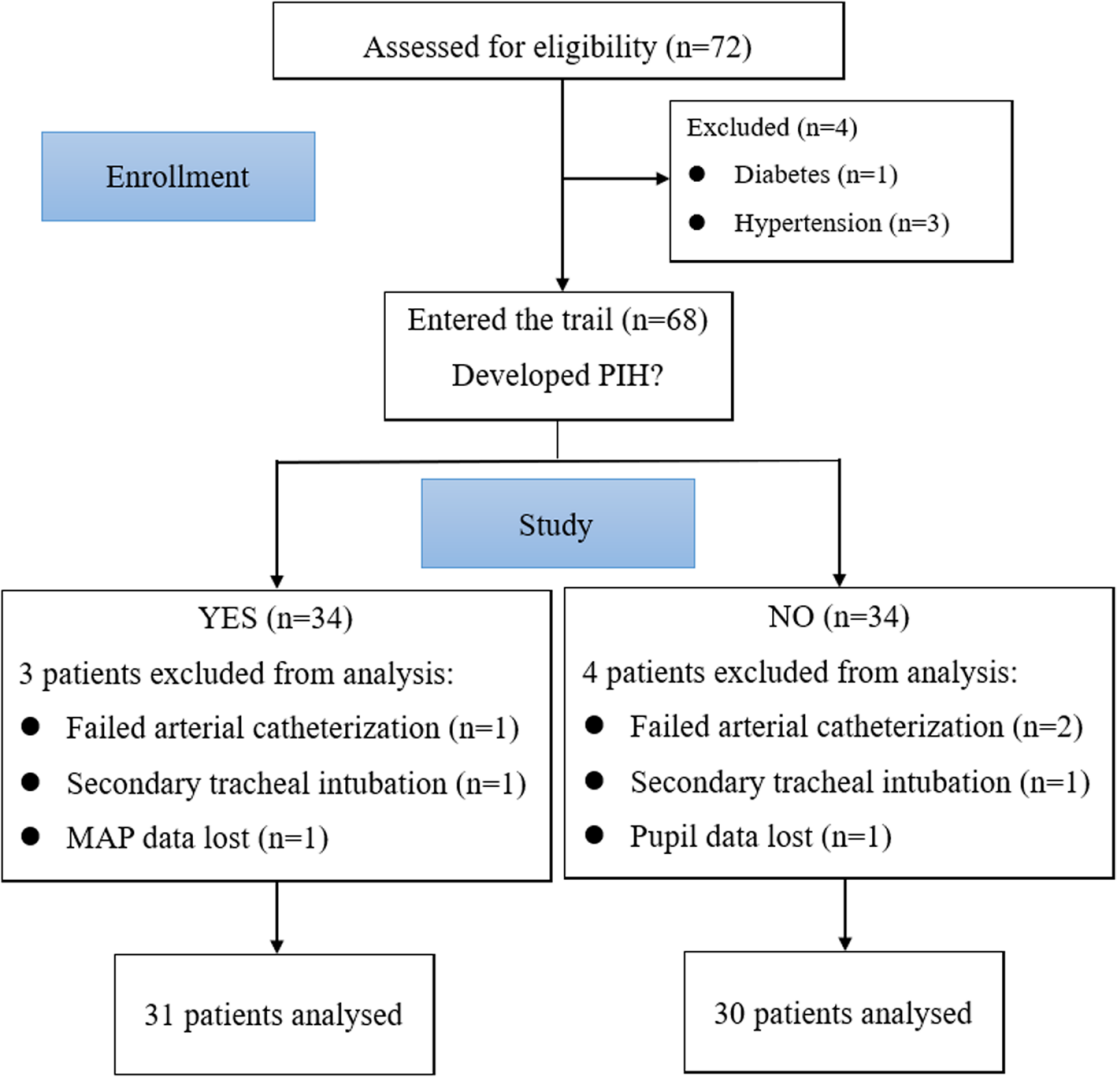
Study flow chart

In total, 72 patients were initially included in this study, of whom 4 with diabetes and hypertension were excluded. In addition, 7 patients withdrew from this study including 2 that underwent secondary intubation, 3 failed arterial catheterization, 1 with MAP data lost, and 1 for whom pupillary measurement data were missing. Ultimately, data from 61 patients were analyzed, of whom 31 (50.8%) developed PIH. Surgical procedures included gynecological (*n* = 8), general (*n* = 14), otolaryngology (*n* = 17), plastic (*n* = 5), urology (*n* = 12), and orthopedic procedures (*n* = 5) (Fig. 2).

### Patient data

Of the 50.8% (31/61) of enrolled patients that developed PIH, 23 exhibited a MAP of < 65 mmHg, while 24 had a > 30% reduction in MAP. There were no significant differences between patients that did and did not develop PIH with respect to preoperative red blood cell counts, hemoglobin, hematocrit, propofol, sufentanil, baseline MAP, baseline HR, or percentage change in HR. Patients in the PIH group, however, were older than those in the non-PIH group and exhibited lower preoperative albumin levels (Table 1).

**Table 1:** Patient baseline characteristics

	PIH (*n* = 31)	no-PIH (*n* = 30)	*P*
Age overall (years)	42 [29–51]	31 [24–36]	0.007
18–24	3 (9.7)	8 (26.7)	
25–34	8 (25.8)	13 (43.3)	
35–44	6 (19.4)	6 (20.0)	
45–54	12 (38.7)	2 (6.7)	
55–64	2 (6.5)	1 (3.3)	
Gender (M/F)	15/16	17/13	0.517
BMI, kg/m^2	23.06 ± 3.11	23.78 ± 3.64	0.409
ASA (I/II)	3/28	3/27	1.000
History of smoking	7 (22.6)	9 (30.0)	0.510
History of drinking	8 (25.8)	7 (23.3)	0.823
Sinus bradycardia	6 (19.4)	3 (10)	0.504
Sinus arrhythmia	1 (3.2)	0 (0)	1.00
Left ventricular high voltage	1 (3.2)	1 (3.3)	1.00
Aortic sclerosis	3 (9.7)	0 (0)	0.248
Red blood cell, 10^12/L	4.47 [3.98–4.97]	4.40 [4.21–4.91]	0.708
Hemoglobin, g/L	136.0 ± 16.9	136.3 ± 15.8	0.949
Hematocrit	0.404 [0.368–0.442]	0.402 [0.373–0.431]	0.708
Albumin, g/L	42.0 ± 3.0	43.7 ± 3.4	0.046
Propofol, mg	124 [110–140]	141 [106–156]	0.191
Sufentanil, μg	19.0 [16.5–21.0]	21.2 [16.0–23.0]	0.157
Baseline MAP, mmHg	97 ± 13	95 ± 11	0.470
Baseline HR, beats/min	76 ± 11	75 ± 13	0.749
Decrease in MAP (%)	34.9 ± 8.0	20.7 ± 7.1	< 0.001
Percentage change in HR(%)	13.9 ± 10.9	9.8 ± 18.3	0.598

*BMI* Body mass index, *ASA* American Society of Anesthesiologists physical status, $Decrease\ in\ MAP=\left(\frac{Baseline\ MAP- Minimum\ MAP}{Baseline\ MAP}\right)\times 100\%$

$$Percentage\ change\ in\ HR=\left(\frac{Baseline\ HR- HR\ at\ minimum\ MAP}{Baseline\ HR}\right)\times 100\%$$

Normally distributed results were reported as means ± standard deviation ($\overline{x}$ ± s), while non-normally distributed data were reported as the median [interquartile range]. Categorical data were reported as numbers (%)

### Pupillary light reflex data

**Fig. 3:**
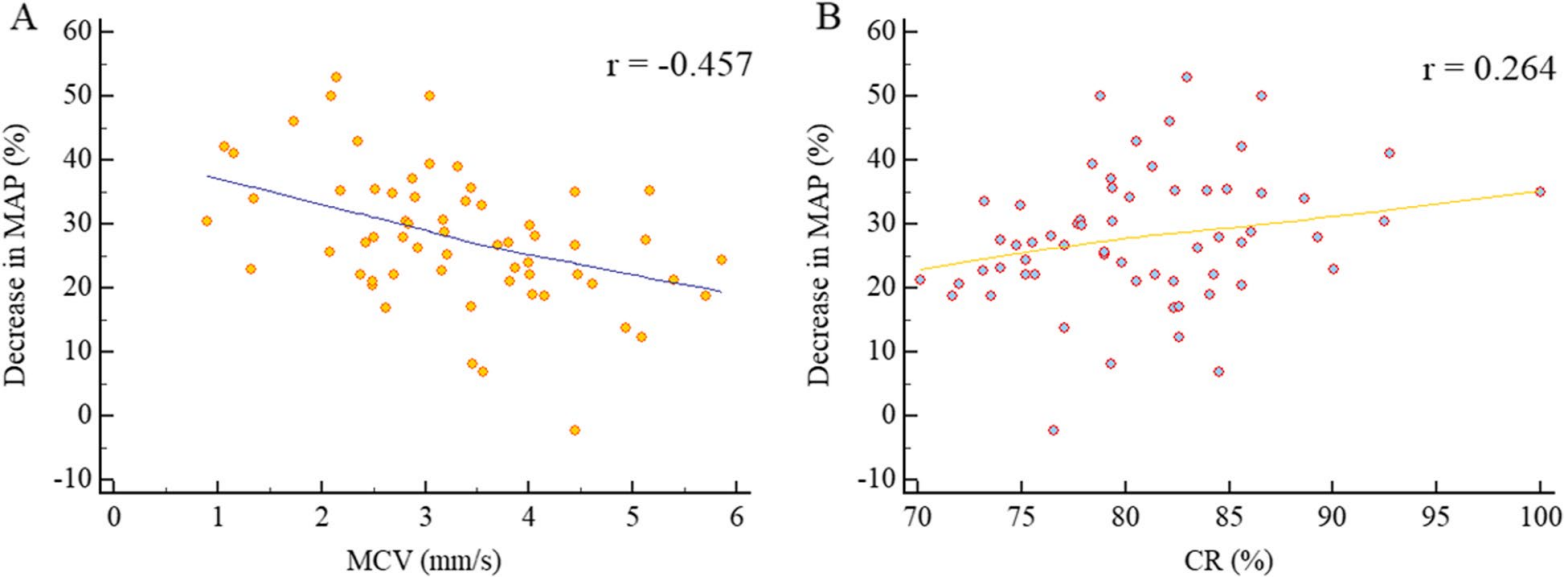
Scatter plots demonstrating the associations between MCV and CR with decreases in MAP relative to baseline following general anesthesia induction. MCV: Maximum Constriction Velocity, CR: Constriction Ratio (%), $CR=\left(\frac{MPD}{BPD}\right)\times 100\%$

Relative to patients unaffected by PIH, individuals in the PIH group exhibited smaller ACV (*P* = 0.003) and MCV values (*P* < 0.001), as well as a higher CR (*P* = 0.003) (Table 2). Decreases in MAP were negatively correlated with MCV (*r* = − 0.457; *P* < 0.001), while they were positively correlated with CR (*r* = 0.264; *P* = 0.040) (Fig. 3).

**Table 2:** Pupil measurement comparisons

	PIH (*n* = 31)	no-PIH (*n* = 30)	*P*
BPD, mm	3.946 [3.415–4.387]	4.166 [3.679–4.752]	0.286
MPD, mm	3.248 [2.756–3.709]	3.300 [2.998–3.538]	0.931
PCL, s	0.200 [0.167–0.233]	0.184 [0.133–0.300]	0.483
PCT, s	0.433 [0.400–0.500]	0.417 [0.392–0.500]	0.618
ACV, mm/s	1.696 ± 0.577	2.118 ± 0.474	0.003
MCV, mm/s	2.736 ± 1.128	3.855 ± 0.882	< 0.001
1/MCV, s/mm	0.372 [0.302–0.476]	0.260 [0.224–0.314]	< 0.001
CR, %	82.841 ± 5.903	78.602 ± 4.762	0.003

*BPD* Baseline Pupil Diameter, *MPD* Minimum Pupil Diameter, *PCL* Pupil Constriction Latency, *PCT* Pupil Constriction Time, *ACV* Average Constriction Velocity, $ACV=\frac{BPD- MPD}{PCT}$; *MCV* Maximum Constriction Velocity, 1/MCV the reciprocal of MCV, CR Constriction Ratio (%),$CR=\left(\frac{MPD}{BPD}\right)\times 100\%$

Normally distributed results were reported as means ± standard deviation ($\overline{x}$ ± s), while non-normally distributed data were reported as the median [interquartile range]. Categorical data were reported as numbers (%)

### Regression analysis

Univariate analyses revealed a relationship between PIH and ACV, MCV, and CR. Following the adjustment of these results for age (Model 1), all three of these variables remained correlated with PIH. When these results were further adjusted for age, sex, ASA physical status, albumin levels, BMI, and baseline MAP (Model 2), both MCV (OR 0.369, 95%CI 0.067–0.866; *P* = 0.008) and CR (OR 1.185, 95%CI 1.043–1.346; *P* = 0.009) were identified as significant independent predictors of PIH, while ACV was weakly associated with PIH (OR 0.240, 95%CI 0.067–0.866; *P* = 0.029) (Table 3).

**Table 3:** Predictive factors for PIH

Predictors	Unadjusted analysis OR [95% CI]	Adjusted analysis OR [95% CI]	
		Model 1	Model 2
ACV, mm/s	0.210 [0.068–0.646] **	0.275 [0.086–0.879] *	0.240 [0.067–0.866] *
MCV, mm/s	0.328 [0.171–0.628] **	0.382 [0.193–0.756] **	0.369 [0.177–0.772] **
CR, %	1.171 [1.045–1.312] **	1.175 [1.044–1.323] **	1.185 [1.043–1.346] **

* *P* < 0.05, ** *P* < 0.01. ACV Average Constriction Velocity, $ACV=\frac{BPD- MPD}{PCT}$, MCV Maximum Constriction Velocity and CR Constriction Ratio (%), $CR=\left(\frac{MPD}{BPD}\right)\times 100\%$

Model 1: Adjusted for age

Model 2: Adjusted for age, sex, ASA physical status, albumin levels, BMI, and baseline MAP

### ROC curve analyses

**Fig. 4:**
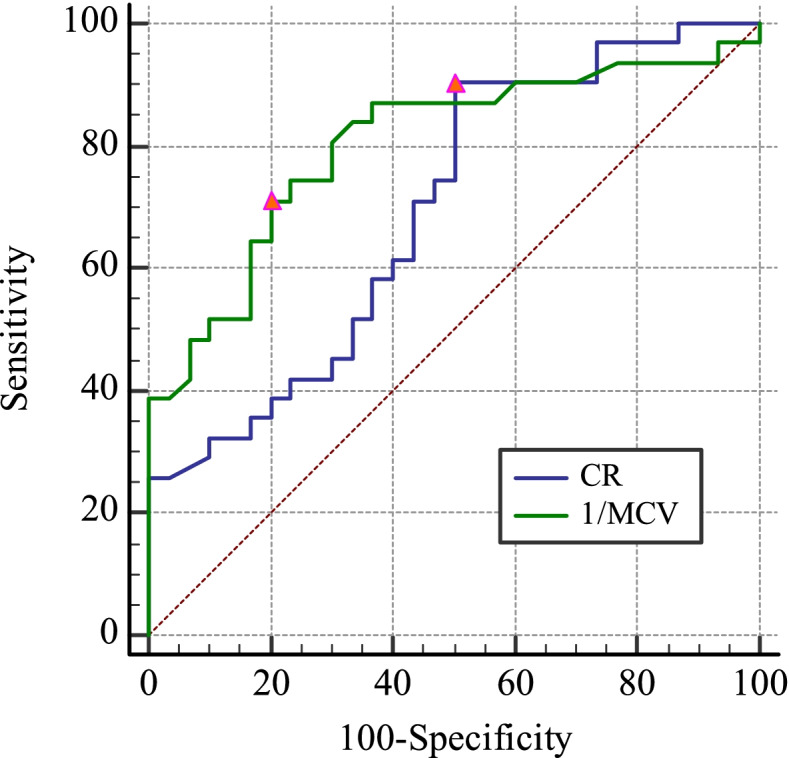
Receiver operating characteristic curves analyses of the diagnostic utility of 1/MCV and CR as predictors of PIH incidence. Triangles corresponding to optimal cutoff values are established based on maximum Youden’s index values

Based on the results of logistic regression, we analyzed the predictive power of the MCV and CR for PIH. Given that MCV was a protective factor with respect to PIH incidence, ROC curve analyses were performed based on the reciprocal of MCV (1/MCV). 1/MCV exhibited good diagnostic value when used as a predictor (AUC: 0.803; 95% CI: 0.681–0.894; *P* < 0.001), with the optimal 1/MCV cutoff value (0.316 s/mm) yielding respective sensitivity and specificity values of 70.97, and 80.00%. Relative to 1/MCV, CR exhibited relatively poor diagnostic accuracy (AUC: 0.695; 95% CI: 0.563–0.806; *P* = 0.004), with an optimal CR cutoff value of 77.05 yielding respective sensitivity and specificity values of 90.32 and 50.00% (Fig. 4).

## Discussion

The results of this study revealed that PLR testing prior to general anesthesia induction can aid in the identification of patients at an elevated risk of developing PIH. In this analysis, MCV was found to offer greater predictive value, with an AUC of 0.803 (0.681–0.894) and respective sensitivity and specificity values of 70.97 and 80.00% at an optimal cutoff value of 0.316 s/mm. Relative to MCV, CR exhibited relatively poor diagnostic accuracy (AUC: 0.695; 95% CI: 0.563–0.806), yielding the weak relationship with decreases in MAP. As a potential tool for predicting PIH, the predictive ability of CR for PIH was less reliable.

The incidence of PIH may be related to abnormal autonomic nervous system functionality or increased sympathetic nervous system activity [10, 20]. Both ACV and MCV can be leveraged as indices for parasympathetic nervous system regulation, with the latter being more sensitive in this context [13]. Parasympathetic nervous system excitation generally results in the slowing of the heart rate and a reduction in blood pressure. In this study, however, patients with higher MCV values were found to be at a lower risk of developing PIH. This may be attributable to the fact that patients with hypertension and diabetes, who may exhibit impaired autonomic nerve function, were excluded from these analyses. Higher MCV values among patients with normal autonomic function may thus correspond to a greater degree of autonomic regulation. Consistently, Muppidi et al. previously reported that pupil constriction velocity was more rapid in patients with normal autonomic nerve function relative to patients with impaired autonomic nerve function. The better autonomic nerve function may enable appropriate hemodynamic regulation in patients following anesthesia induction, thus preventing PIH [11]. CR is an index for the function of the sympathetic nervous system, and is positively correlated with LF/HF, which corresponds to the status of the sympathetic-parasympathetic system [13]. Here, those patients exhibiting higher preoperative CR values corresponding to greater sympathetic nervous system tension were found to be at a greater risk of developing PIH, in line with prior data indicating that patients with higher LF/HF were more likely to develop PIH [6]. The drugs used for anesthesia induction, particularly propofol and sufentanil, inhibit the function of the sympathetic system, leading to reductions in heart rate and blood pressure. In patients with a preoperative state of sympathetic tension, more extreme hemodynamic fluctuations may occur following induction, thus contributing to PIH incidence.

In illuminated settings, resting pupil size is under the control of the sympathetic and parasympathetic nervous system, with the parasympathetic system being the primary regulator of the velocity and magnitude of pupil constriction upon light stimulation [15]. When such stimulation is discontinued, pupil dilation is under the control of the central and peripheral sympathetic nervous system [21]. Dynamic pupillary testing has emerged as an approach to assessing autonomic neuropathy in patients with diabetes [22, 23]. While direct observation of the pupil can only yield qualitative results regarding sensitivity/insensitivity and the presence or absence of a response to stimuli, the use of an infrared pupil camcorder paired with a computer with appropriate analytical software was sufficient to enable the rapid and objective measurement of quantitative PLR parameters. By analyzing the parameters associated with separate phases of the PLR process, it is then possible to evaluate autonomic nervous function accurately and comprehensively in a given patient. In this study, all pupillometric measurements were made from 9 AM – 12 AM to exclude any impact of diurnal variations on the resultant data [24]. As such, PLR testing performed prior to general anesthesia induction represents an effective means of gauging autonomic nervous function and thereby identifying individuals at a higher risk of PIH.

In this analysis, older patients were found to be at a higher risk of PIH, consistent with the results of a large retrospective study conducted by Riech et al. in which individuals > 50 years old were at a 2.25-fold higher risk of PIH relative to younger patients [5]. Even so, age was not identified as an independent predictor of PIH status in this study following logistic regression analyses using Model 2. This may be attributable to the limited sample size in this study or the fact that patients over 64 years of age were excluded from this trial. In follow-up trials, additional patients will thus be incorporated to facilitate a more robust stratified analysis aimed at ensuring these results can be generalized.

Relative to prior studies, the present study is innovative in several respects. First, autonomic neuropathy is a common diabetic complication that is important in the context of PIH development [25]. As such, patients with hypertension and diabetes were excluded from this study to avoid any potential impact of these comorbidities on the results of this analysis. Second, the selected induction protocol is routinely used in our country, with propofol and sufentanil being selected for use in order to maximize the generalizability of these results. Third, in contrast to other studies that have employed noninvasive approaches to monitoring blood pressure, all patients in this study underwent invasive arterial blood pressure monitoring, thus ensuring that continuous, accurate blood pressure data were collected in real-time. Fourth, while one prior study identified a link between dynamic pupillometry and PIH [14], their analyses did not take the potential impact of multiple endotracheal intubations on blood pressure into account, and failed to analyze the forecast effect, with the present study thus addressing these gaps in knowledge.

In this study, PIH affected 50.8% of included patients, in line with the overall observed PIH incidence in a survey of our hospital. In a previous study, Bijker et al. summarized 140 definitions of induced hypotension [26]. For the present study, a more complete definition was used than in many other articles, with PIH being clearly defined as a > 30% reduction in MAP or any recorded MAP < 65 mmHg for at least 1 min during the interval between induction and 10 min following intubation. The selected 65 mmHg threshold is a blood pressure level that is routinely considered tolerable in perioperative and intensive care settings, while the 1-minute time limit avoids transient blood pressure drop caused by improper operation or sensor failure and ensures that anaesthesiologists promptly treat the hypotensive episode in an effort to prevent any further drop in blood pressure or associated postoperative complications [27–29]. The definition of the induction period as the interval between induction and 10 minutes post-intubation was selected to avoid major changes in blood pressure as a consequence of additional stimulation.

There are several limitations to this study. Firstly, we only included patients who had no autonomic dysfunction. Further research on the relationship between pupil parameters and PIH in people with diabetes or hypertension who may also have autonomic dysfunction is necessary. Repeated assessments on individuals and a broader cohort would be beneficial to confirm the validity of our results. Secondly, an essential component of the study is the definition of hypotension. Our composite definition of PIH has several limitations, and hypertensive individuals should not use MAP < 65 mmHg. The experiment will be better and more accurate when only patients who have experienced a clear and significant fall in MAP are included rather than those who only experienced a minor decline. Thirdly, while all patient PLR measurements were taken under the same light, the larger space of the anesthesia preparation room may have led to an impact of external ambient light on study results [30]. Finally, some patients undergo uncontrollable blinking following PLR, which can impact measurements of sympathetic parameters including pupil dilation velocity and dilation latency.

## Conclusion

PIH is a common complication of general anesthesia induction that is related to more advanced age, lower MCV, and higher CR. Compared with CR, the measurements of MCV in the preparation room before anesthetization can better predict PIH incidence in patients of ASA I-II status. Further research should thus focus on validating the results of this analysis in a larger patient cohort.

## Data Availability

The datasets used and/or analysed during the current study are available from the corresponding author on reasonable request.

## Abbreviations

PLR: Pupillary light reflex
PIH: Post-induction hypotension
ASA: American Society of Anesthesiologists physical status
BMI: Body mass index
MAP: Mean arterial pressure
HR: Heart rate
BPD: Baseline pupil diameter
MPD: Minimum pupil diameter
PLC: Pupil constriction latency
PCT: Pupil constriction time
ACV: Average constriction velocity
MCV: Maximum constriction velocity
CR: Constriction ratio
CO_2_: Carbon dioxide
ROC: Receiver operating characteristic
